# A blended module (STAIRS) to promote functional and personal recovery in patients with a major depressive disorder in remission: study protocol of a concurrent mixed methods randomized controlled trial

**DOI:** 10.1186/s12888-023-05213-w

**Published:** 2023-10-07

**Authors:** David Wedema, Klaas J. Wardenaar, Manna A. Alma, Antoinette D. I. van Asselt, Eliza L. Korevaar, Robert A. Schoevers

**Affiliations:** 1 Research and Innovation Centre for Rehabilitation, Hanze University of Applied Sciences, Groningen, The Netherlands; 2 Department of Behavioural and Social Sciences, University of Groningen, Groningen, The Netherlands; 3 Department of Health Sciences, Applied Health Research, University of Groningen, University Medical Centre Groningen, Groningen, The Netherlands; 4 Department of Epidemiology, University of Groningen, University Medical Centre Groningen, Groningen, The Netherlands; 5 Department of Psychiatry, Research School of Behavioural and Cognitive Neurosciences (BCN), University of Groningen, University Medical Centre Groningen, Groningen, The Netherlands

**Keywords:** Functional recovery, Personal recovery, Depression, Peer group, Expert by experience, Blended care, Social support, Recovery-oriented care

## Abstract

**Background:**

Despite the availability of a wide variety of evidence-based treatments for major depressive disorder (MDD), many patients still experience impairments in their lives after remission. Programs are needed that effectively support patients in coping with these impairments. The program Storytelling and Training to Advance Individual Recovery Skills (STAIRS) was developed to address this need and combines the use of peer contact, expert-by-experience guidance, family support and professional blended care. The aim of the planned study is (1) to assess the efficacy of the STAIRS program in patients with remitted MDD, (2) to investigate patients’ subjective experiences with STAIRS, and (3) to evaluate the program’s cost-effectiveness.

**Methods:**

A concurrent mixed-methods randomized controlled trial design will be used. Patients aged between 18 and 65 years with remitted MDD (*N* = 140) will be randomized to either a group receiving care as usual (CAU) + the STAIRS-program or a control group receiving CAU + some basic psychoeducation. Quantitative efficacy data on functional and personal recovery and associated aspects will be collected using self-report questionnaires at the start of the intervention, immediately following the intervention, and at the six-month follow-up. Insights into patients’ experiences on perceived effects and the way in which different program elements contribute to this effect, as well as the usability and acceptability of the program, will be gained by conducting qualitative interviews with patients from the experimental group, who are selected using maximum variation sampling. Finally, data on healthcare resource use, productivity loss and quality of life will be collected and analysed to assess the cost-effectiveness and cost-utility of the STAIRS-program.

**Discussion:**

Well-designed recovery-oriented programs for patients suffering from MDD are scarce. If efficacy and cost-effectiveness are demonstrated with this study and patients experience the STAIRS program as usable and acceptable, this program can be a valuable addition to CAU. The qualitative interviews may give insights into what works for whom, which can be used to promote implementation.

**Trial registration:**

This trial was registered at ClinicalTrials.gov on 1 July 2021, registration number NCT05440812.

**Supplementary Information:**

The online version contains supplementary material available at 10.1186/s12888-023-05213-w.

## Background

Major depressive disorder (MDD) is a highly prevalent and recurring disorder with a strong negative impact on patients, their family members and society as a whole [1–3]. Epidemiologic studies show that approximately 5% of the adult general world population is currently suffering from MDD, which affects approximately 280 million people, making it a leading cause of disability [4]. Treatments to effectively address the negative impact of depression are therefore highly needed.

The acute treatment of MDD symptomatology usually consists of prescription of antidepressant medication and psychological interventions, often in combination [5]. Previous research has shown that these treatments are moderately effective and that 50 to 60% of patients do not achieve an adequate response to treatment [6–9]. Importantly, of the patients, who are considered as remitted from MDD based on their symptom severity, more than half do not subjectively consider themselves to be in full remission, as they still experience functional impairments, deficits in their coping ability and lower quality of life [10]. The collective results from most treatment studies thus represent a rather narrow approach to the concept of recovery, as they have exclusively focused on evaluating treatment effects on the presence and severity of symptoms [11]. This does not necessarily align well with patients’ subjectively experienced recovery process. In fact, in their desired treatment outcome, patients place an emphasis on aspects such as finding new ways of daily functioning, feeling in control again, regaining a sense of meaning in life and the ability to undertake activities with their loved ones again, in addition to symptom reduction [12–14].

To address the aspects that are seen as important by patients themselves, two domains have been added to the concept of recovery in the past decades by community-oriented social psychiatry and the consumer movement (e.g., the antipsychiatry movement and the disability rights movement). The first domain focuses on restoring functioning after depression and is referred to as *functional recovery*. In this domain, the focus lies on enabling patients to manage tasks at work/school, engage in meaningful relationships and perform daily activities [15]. The second domain focuses on redefining a patient’s identity and is referred to as *personal recovery*. A widely used definition of personal recovery is that of Anthony [16], describing it as a unique and personal process in which patients find a new balance in life where they are satisfied, hopeful and feel they have a meaningful life, even when there remain limitations caused by an illness. The key aspect of personal recovery is the ability of patients to find a new identity, in which they feel in control again and have hope for the future. Both functional and personal recovery relate to patients’ well-being and are therefore included in a broader view on recovery, which has been adopted in MDD treatment guidelines [5]. This broader view on recovery fits in with the shift in mental healthcare towards an approach in which the patient perspective is given more attention and health is seen as more than the absence of physical, mental and social obstacles [17].

The three domains of recovery (clinical, functional and personal) complement each other but do not necessarily develop simultaneously during the recovery process [18–21]. Being in symptomatic or clinical remission does not inherently mean that someone has found a new balance in life in which they can cope with the difficulties resulting from the illness. Functional and personal recovery can lag behind clinical recovery by up to several years [22–24]. One reason for this is a stronger feeling of incompetence caused by identifying oneself with the mental illness (illness identity), which has an impact on hope and self-esteem and in turn can negatively influence coping with daily tasks, social interaction and vocational outcomes [25]. Lasting effects in the functional and personal domains are known risk factors for symptomatic relapse [15, 26]. Consequently, interventions aimed at enhancing personal and functional recovery may also contribute to perpetuating clinical recovery and preventing relapse into new depressive episodes.

The above shows that we should ideally pay more attention to personal and functional recovery in depression treatment to optimise treatment outcome in MDD patients. However, current treatment guidelines are predominantly based on efficacy studies in which outcome is defined as clinical recovery [11, 27]. One barrier to adopting a broader approach to recovery in depression treatment may be that comprehensive and well-documented programs focused on functional and personal recovery in MDD patients are currently lacking. Recovery-oriented treatments have their origin in the field of schizophrenia treatment, where complete symptomatic recovery is rare due to the chronic nature of mental problems. Therefore, to enable patients to live with their symptoms and reach or maintain an acceptable quality of life, treatment is often focused on patients’ identity and on helping patients optimize their situation in other domains (e.g., social relations, work) [28]. To the best of our knowledge, such an approach has not yet been adopted in depression treatment. As many patients suffering from MDD also experience residual symptoms after remission [29] and a long-lasting impact of the illness on their identity and daily and social functioning (e.g., managing tasks at work/school and engaging in meaningful relationships) [30], recovery-oriented treatment could also be valuable for them. This type of treatment is the focus of this study.

For treatment to promote personal recovery in mental health, Leamy et al. [31] proposed a conceptual framework, in which five recovery processes are described: (1) *connectedness* with others, (2) *hope* that there are possibilities to get better, (3) a redefined *identity* wherein there is balance between vulnerabilities and possibilities, (4) *meaning* to life (including the ability to fulfil significant roles in life) and (5) a sense of *empowerment*. These processes fit well with the nine elements of functional and personal recovery described in an earlier publication by Davidson et al. [32]: (1) renewing hope and commitment, (2) redefining self, (3) incorporating illness, (4) being involved in meaningful activities, (5) overcoming stigma, (6) assuming control, (7) becoming empowered and exercising citizenship, (8) managing symptoms and (9) being supported by others [32]. Taken together, the aspects described by Leamy et al. [31] and Davidsons et al. [32] are widely used in recovery-oriented research and form the theoretical basis for the current work. Here, the assumption is that the stronger a treatment influences these aspects, the more likely it is to contribute to functional and personal recovery. Previous research has found several elements that can be incorporated into treatment and that support one or more of these aspects. *Peer contact* has been found to promote social contact, mutual understanding, trust and self-reliance [33–35]. G*uidance by an expert by experience* has been found to raise empowerment and a feeling of hope, to improve social functioning, and to reduce stigma and self-stigma [36–38]. *Family support* has been shown to promote hope and a more positive sense of self [39, 40] and to function as a buffer against stressful life events [41]. In addition, the use of online facilities next to face-to-face contact (*blended care*) has been shown to enhance self-management, autonomy and connectedness [42–44]. To the best of our knowledge, no program that combines all of these elements to support recovery in MDD patients has previously been developed.

To address this gap, the training program ‘Storytelling and Training to Advance Individual Recovery Skills’ (STAIRS) [45] was developed by a project team (consisting of clinical professionals and experts by experience). In the STAIRS program, all of the above-described elements are integrated. Furthermore, the program is designed to foster the mechanisms that were concluded to be supportive of recovery in a recent systematic review [46]: (1) provision of information and teaching skills, (2) promotion of a working alliance, (3) the use of role modelling and (4) increasing choice and opportunities. To evaluate the STAIRS program’s efficacy in improving recovery, we aim to conduct a randomized controlled superiority trial (RCT) among MDD patients, who are in symptomatic remission. The intervention group will receive STAIRS training on top of care as usual (CAU). The comparison group will receive CAU and basic written psychoeducation about the recovery process. The primary quantitative outcome of the RCT is a measure of functional and personal recovery. Secondary outcomes include measures of depressive symptom severity, experienced control over life and cost-effectiveness. In exploratory quantitative analyses, potential demographic moderators of responding to the STAIRS-training will be investigated. Our primary hypothesis is that patients assigned to the STAIRS group will reach a significantly higher level of functional and personal recovery than the patients assigned to the comparison group. In addition, we hypothesize that the STAIRS group will show: (1) lower symptom severity and relapse rates in the first six months following the intervention, (2) lower levels of depression-related functional disabilities, (3) higher levels of perceived empowerment, (4) higher levels of perceived control of events and ongoing situations, (5) higher levels of perceived self-management skills, and (6) lower costs per quality-adjusted life year (QALY) compared to the comparison condition.

## Methods/design

### Study design

**Fig. 1:**
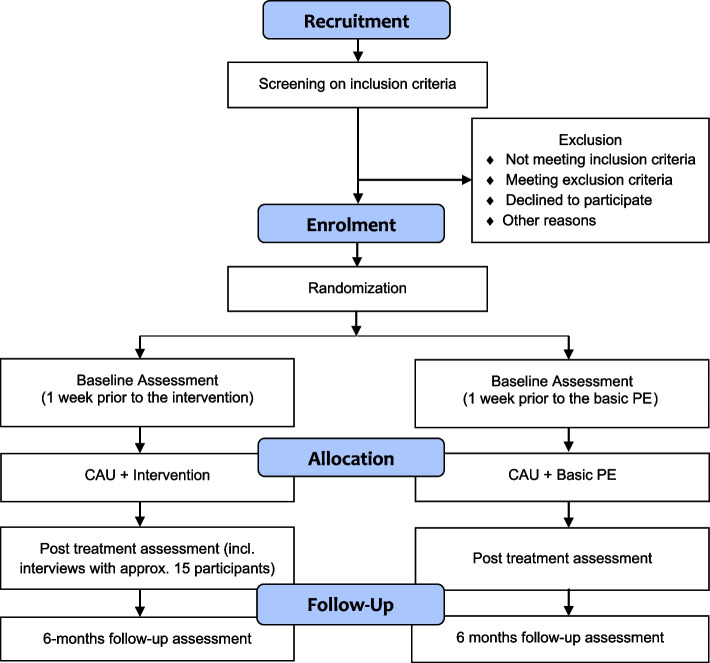
CONSORT flowchart of the study; CAU = care as usual; PE = psychoeducation

We will use a concurrent mixed methods randomized controlled superiority trial [47], comparing the STAIRS-program to CAU (with some psychoeducation) on a range of quantitative outcomes. Additionally, a qualitative study will be conducted with a subgroup of the study sample to gain more insight into the mechanisms underlying the quantitative study outcomes. Combining both types of data makes it possible to better interpret what contributes in which way for whom. This is particularly important since the intervention under study consists of a combination of aspects that interact with each other [48]. See Fig. 1 for an illustration of the design.

The SPIRIT checklist is presented as an additional file and describes where the items described in the SPIRIT reporting guidelines [49] are located in this protocol. After drawing up the first protocol (approved by the Medical Ethical Committee; METc, on 21–12-2021), three amendments have been made and submitted to the METc. Amendment no. 1 (21–2-2022) concerned an adjustment in the control condition. The previous control condition deviated too much from CAU and was insufficiently in line with the phase of recovery in which the target group for this study is located. Amendment no. 2 (30–1-2023) concerned an adjustment to the inclusion criterion aimed at the phase of the treatment, in order to better align with this treatment and achieve more inclusions. A demarcation in time (i.e. the last six months of treatment) turned out to be insufficiently in line with how depression treatment proceeds in practice and has therefore been changed to a demarcation in terms of reduction of depression symptoms (i.e. a decrease in symptom severity to moderate or less). The last amendment (22–6-2023) concerned the addition of the possibility to also offer the STAIRS training online so that patients for whom physical meetings are not possible can still participate.

### Recruitment and randomization

Participants are recruited from October 2022 to June 2024 from several mental healthcare organizations in the North of the Netherlands (including the University Center of Psychiatry [UCP] from the University Medical Center Groningen [UMCG], Mental Health Services Drenthe [GGZ-Drenthe], and Dimence).

Patients can be included in the study if they are between 18 and 65 years old, receive treatment for MDD and have depressive symptom severity levels that have decreased to moderate or less, according to the Inventory of Depressive Symptomatology self-report (IDS-SR). Exclusion criteria are: a primary diagnosis of bipolar depression or depression with psychotic features, comorbid schizophrenia spectrum or other psychotic disorder, comorbid moderate or severe dependence on alcohol or drugs, and neurological disorders (e.g., dementia). Additionally, patients with insufficient command of the Dutch language, cognitive problems or indication of low IQ (i.e., < 80), who do not possess a computer or smartphone, and/or who have been referred to a different mental health service for other mental problems are excluded.

In the participating centres, treating clinicians will inform eligible patients about the study and provide contact information to the coordinating researcher (DW) when a patient is willing to participate. Also, information about this study will be shared on different social media platforms, allowing interested participants to contact the coordinating researcher themselves. Once a patient has expressed their interest in participating, a researcher will contact them to further explain the study and check eligibility. An information letter will be sent, and a patient will have a week to contemplate, after which they will be contacted again. When a patient is willing to participate, a meeting will be planned with a researcher to discuss any further questions and to sign an informed consent form.

After informed consent is obtained, patients will be randomly allocated to one of the study arms. Randomization will be performed at the patient level by a research assistant who is not involved in the study, using simple randomization with a 1:1 allocation ratio. A list with 140 randomly generated numbers (0 or 1) will be used to allocate patients in order of the date and time of the informed consent meeting. Patients who are randomized to the active intervention condition will be placed on a waiting list for the treatment in (a) the organization where they received all their previous treatment (for patients referred from organizations that offer the STAIRS training) or (b) in the UCP (for patients who are referred from organizations that do not offer the STAIRS training). The STAIRS training starts when at least three patients have been added to the waiting list. At that time, patients will receive an email inviting them to join the training. This email will contain all needed information about the date and location of the meetings and how to access the private online environment that is used as part of the STAIRS-program. When attending physical meetings turns out to be difficult for participants in the intervention condition, they will be given the option to follow the STAIRS training online. Previous pilot work showed this to be a usable and acceptable option [45].

### Interventions

All patients who are included in the RCT will continue to receive CAU according to applicable clinical guidelines during the complete runtime of the study. CAU can consist of psychotherapeutic and/or drug treatment, outpatient, day or inpatient treatment and individual or group treatment.

The main goal of the STAIRS program is to increase the level of functional and personal recovery of patients suffering from the lasting impact of depression on their lives. This will be done with different exercises, by sharing experiences and by providing supportive information. The STAIRS training has a duration of eight weeks, in which participants address the following themes in weekly group sessions: effects of depression and treatment, structure, public stigma and self-stigma, self-image, meaningfulness, connecting to others, relaxation and preventing relapse. Each session will be guided by a clinician (psychologist, nurse practitioner or medical social worker) and a certified expert by experience. Trainers will undergo a 1-day train-the-trainer where the aim of the program and the working methods used are discussed, the complete program is reviewed and example exercises are practised. In between sessions, participants can keep working on a theme via homework assignments on which they will be asked to reflect and interact with each other in a private online community.

Patients in the control condition will receive CAU and some basic psychoeducation. The latter will be delivered in three different information letters (see Additional file 1), which the patients subsequently receive one, four and seven weeks after completing the baseline questionnaires. Each information letter focuses on one of three main aspects of the recovery process: (1) regaining structure in daily life, (2) redefining social relationships and (3) finding a new balance in life. The letters provide basic information about the recovery process, tips on how to cope with depression in everyday life and links to videos, in which peers share experiences regarding the role of the described aspect in their recovery process. The purpose of these letters is to offer practical information on strategies supporting the recovery process, which patients can use as they see fit in their own situation. We find that this intervention can be regarded as a slightly augmented form of CAU. This approach was chosen in favour of delivering only CAU in this group because patients may end their MDD treatment during the study. The psychoeducation letters will offer us the opportunity to keep informing and involving patients and to keep them engaged with the study, even after ending their treatment. At the same time, the added elements are minimal, making sure that the comparison condition is still very close to CAU as it is currently offered in practice.

### Measurements

**Fig. 2:**
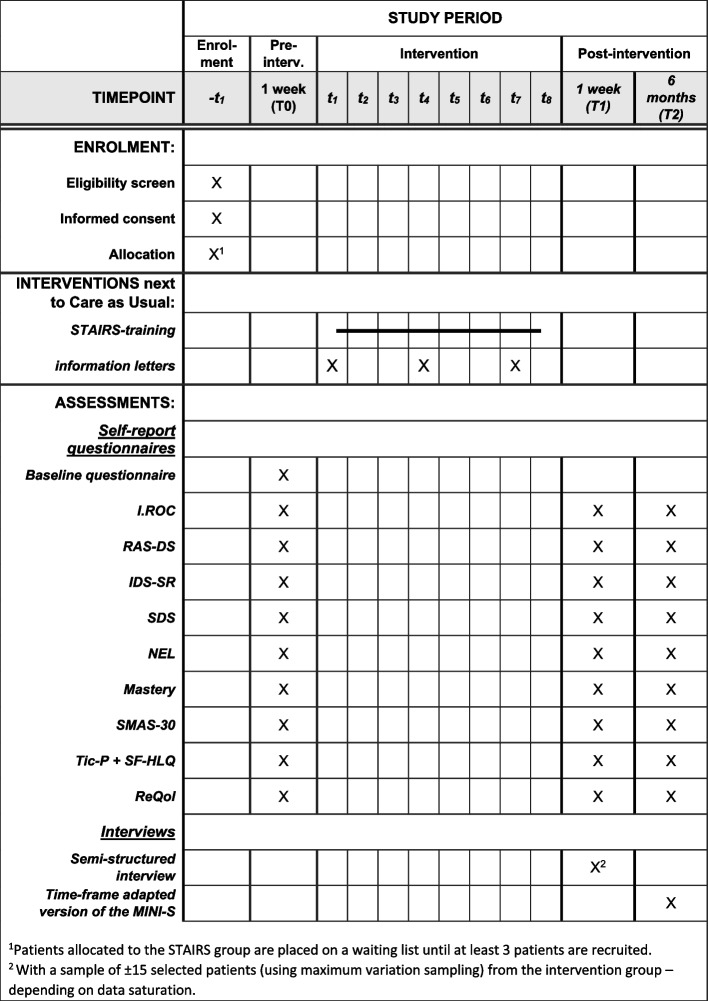
Overview of enrolment, intervention and assessments

Quantitative data will be collected by administering online self-report questionnaires in the week prior to the intervention (T0; baseline), the week following the intervention (T1) and 6 months later (T2). Patients can complete the questionnaires at home. A reminder will be sent after three days, and contact will be made by telephone after a week to promote participant retention. In addition to the questionnaires, a short structured interview will be conducted at T2 to assess depressive relapse in the past 6 months. Qualitative data will be collected by conducting semi structured in-depth interviews with a selection of patients from the intervention group, immediately after the end of the STAIRS-program (T1). Figure 2 gives an overview of the planned enrolment, interventions and assessments.

### Primary outcome measure

#### Recovery

As STAIRS was developed to support recovery beyond clinical recovery, we chose subjective experienced recovery as the primary outcome measure, which entails both functional and personal recovery. This will be measured with the Individual Recovery Outcome Counter (I.ROC) [50], which is a self-report questionnaire specifically developed to measure the subjective experienced recovery of patients. It focuses on four domains of well-being: (1) home, (2) opportunity, (3) people and (4) empowerment. Each domain consists of 3 recovery aspects, which are described with several keywords and a single question to indicate the level of this aspect in one’s life during the last three months (on a 6-point Likert scale ranging from ‘never’ to ‘always’). The I.ROC sum score of all items (range 12–72) will be used to measure the level of recovery, with higher scores indicating better recovery. The domains focused on by the I.ROC cover both aspects of functional recovery (e.g., everyday skills, movement and being active, social network) and personal recovery (e.g., meaning and life goals, self-esteem, hope for the future). The I.ROC has been shown to have good internal consistency with a Cronbach’s alpha of 0.86 and was found to be a preferred recovery measurement tool by patients compared to two other commonly used recovery measures [50].

### Secondary outcome measures

#### Sample characteristics

A baseline questionnaire consisting of 19 questions related to a patient’s demographic characteristics, education/work status and mental/physical health will be used to gain insight into the sample characteristics. In addition, the gathered information can potentially be used as covariates in analyses to investigate the effects of person characteristics on the effect of STAIRS.

#### Recovery

To be able to compare the results internationally, we will also measure recovery with the Dutch version of the Recovery Assessment Scale – Domains and Stages (RAS-DS) [51]. The RAS-DS is a widely used self-report questionnaire containing 38 recovery-oriented statements focusing on four domains: (1) doing things I value, (2) looking forward, (3) mastering my illness, and (4) connecting and belonging. Patients indicate the level of how much each statement reflects their current lives on a 4-point Likert scale (1 = not true, 4 = totally true). The sum score of all items (range 38–152) will be used, with higher scores indicating better recovery. The RAS-DS has been shown to have a high internal consistency with a Cronbach’s alpha of 0.96 and to be sensitive to change over time [52].

#### Depression-related items

*Depressive symptom severity* will be assessed using the Inventory of Depressive Symptomatology Self Report (IDS-SR) [53], which is a 30-item self-report questionnaire (of which 28 items are scored). Each item focuses on one specific aspect of symptomatology in the last week and is scored on a 4-point Likert scale (0 = no change/difficulties, 3 = strong change/difficulties). The sum score of 28 items (range 0–84) will be used, with higher scores indicating higher levels of depressive symptomatology. The IDS-SR has been shown to have high internal consistency (Cronbach’s alpha is 0.92) [54].

*Depression-related functional impairment* describes the level of impairment patients can experience due to the presence of a mental illness. This will be assessed using the 5-item self-report Sheehan Disability Scale (SDS) [55]. Three out of the five items focus on the level of impairment patients experienced in the past week with regard to their work/school activities, family relationships, and social functioning and are rated on a 10-point Likert scale. The sum score of three items (range 0–30) will be used, with higher scores indicating more disability and impairment due to depressive symptoms.

*Relapse* will be assessed at T2 to determine if a patient has relapsed in the period following treatment. Assessment will be performed by a trained interviewer using the ‘Major Depressive Episode’ (MDE) section of the Dutch version of the Mini International Neuropsychiatric Interview – Simplified (MINI-S) [56], which is a short structured diagnostic interview. The time frame will be adapted to enable assessment of relapse between T1 and T2.

#### Level of control

*Empowerment* is an important aspect of recovery [31, 57] and will therefore be assessed separately using the Nederlandse Empowerment Lijst (NEL) [57]. The NEL is a 40-item self-report questionnaire that focuses on six domains: (1) social support (a respecting and accepting attitude from family and friends), (2) professional help (supportive relationship with caregiver), (3) connectedness (being in contact with others), (4) confidence and purpose (being in charge of one’s own life), (5) self-management (having strategies to cope with difficulties), and (6) caring community (being able to share experiences and having a concerned society). Each item consists of a statement in which participants indicate to what extent this applies to their lives (1 = strongly disagree, 5 = strongly agree). The sum score of all items (range 40–200) will be used, with higher scores indicating higher levels of empowerment. The NEL has been shown to have high internal consistency (Cronbach’s alpha is 0.94) [57].

*Experienced control over one’s life* will be assessed using the Dutch version of the 7-item Mastery scale [58]. In this self-report questionnaire, patients respond to how much they agree with seven statements on a 5-point Likert scale (1 = strongly disagree, 5 = strongly agree), reflecting the extent to which they experience control over what happens in their lives. The sum score of all items (range 7–35) will be used, with higher scores indicating a stronger sense of control. We chose to include this outcome because patients mentioned control over their life as one of the most important aspects when defining what recovery entails to them [59]. The Mastery Scale has been found to have high internal consistency (Cronbach’s alpha is 0.81) [60].

*Self-management skills* will be assessed using the 30-item Self-Management Ability Scale (SMAS-30) [61]. The SMAS-30 is a self-report questionnaire focusing on six domains: (1) taking initiative, (2) self-efficacy, (3) investment behaviour, (4) positive frame of mind, (5) variety and (6) multifunctionality. Each domain consists of five questions that are scored on either a 6-point Likert scale (1 = never, 6 = very often; for four domains) or a 5-point Likert scale (1 = absolutely yes, 5 = absolutely no; for two domains). After transformation, each domain has a range of 0–20. The sum score of all domains (range 0–100) will be used, with higher scores being indicative of better self-management skills. The SMAS-30 has been found to have high internal consistency (Cronbach’s alpha is 0.90) [62]. Self-management will be assessed because this has been found to be an important skill that contributes to both the prevention of relapse and coping with the day-to-day consequences of (residual) depressive symptoms [63].

### Cost-effectiveness

*Direct and indirect costs* (medical costs and productivity loss) associated with the psychosocial problems caused by depression will be assessed using the 26-item Treatment Inventory of Costs in Patients with psychiatric disorders (TiC-P) [64], which includes the 11-item Short Form Health and Labour Questionnaire (SF-HLQ) [65]. The first part of the TiC-P contains 15 questions that assess contacts with a range of healthcare providers and medical specialists (yes/no; followed by an assessment of the number of visits). The second part focuses on work loss for both paid and unpaid work, and reduced efficiency at work.

*Quality of life* will be assessed using the 10-item Recovering Quality of Life (ReQol) [66]. In this self-report questionnaire, seven domains are assessed: (1) activity, (2) belonging and relationships, (3) choice, control and autonomy, (4) hope, (5) self-perception, (6) wellbeing and (7) physical health. Each domain is covered by one or two items that are scored on a 5-point Likert scale (0 = none of the time, 4 most or all of the time; negatively worded questions will be scored from 4 to 0). The sum score of all domains (range 0–40) will be used, with higher scores indicating higher levels of recovering quality of life. The Dutch version of the ReQol has a high internal consistency (Cronbach’s alpha is 0.90) [67]. We chose the ReQol because this questionnaire is specifically developed to incorporate patient perspectives on all relevant aspects concerning recovery and because it has better responsiveness: a moderate standardized response means [SRMs] was previously found for the ReQol versus small SRMs for the EQ-5D [66]. The Recovering Quality of Life – Utility Index (ReQoL-UI) will be used to convert the ReQoL sum score into a preference-based index score that is used to calculate QALYs [68].

### Qualitative program evaluation

To gain insights into (1) mechanisms and/or program features that contribute to any experienced effects of STAIRS and (2) the usability and acceptability of the program, patients’ perceived value of the STAIRS-training as a whole and of its different elements will be qualitatively evaluated. For this, semi structured in-depth interviews will be conducted by the coordinating researcher (DW; MSc, male, lecturer at a university of applied sciences and PhD candidate) with selected patients until data saturation is reached. We expect that approximately 15 patients are needed for this. Selection will be performed using maximum variation sampling based on age, sex, ethnicity and number of previous depressive episodes to create maximum variation [69]. The interviews will be held at home or another preferred location of the patient. It will take approximately 90 min and will address the following topics: perceived effects (in general, control, identity, social contact), meetings (in general, activities, trainers, peers, structure), homework assignments and the website used. In an iterative process, the interviews will be supplemented with preliminary results from the RCT and previously conducted interviews, allowing patients to reflect on previous findings. The collection of all qualitative data and reporting of the results will be guided by the COnsolidated criteria for REporting Qualitative studies (COREQ) [70].

### Data management

A data management plan (DMP) is created and made available on DMP online. Data will be collected and managed according to the General Data Protection Regulation (GDPR; in Dutch: Algemene Verordening Gegevensbescherming) and applicable codes of conduct, such as the research code of the UMCG. Confidentiality of participant data will be secured by removing all identifiable data and replacing it with a unique identifier. Only the principal investigator and coordinating researcher will have access to the key file that links the unique identifier to the identifiable data. During the trial, all data will be stored on the research drive of the UMCG. After the trial, the data will be stored for a minimum of 15 years in a secured study-specific folder on the research drive of the UMCG. The deidentified data will be made accessible upon request after the latter is assessed by the principal investigator.

### Ethical statement

All procedures of this study will be conducted in accordance with the principles of the Declaration of Helsinki (latest version 19 October 2013) and in accordance with the Dutch Medical Research Involving Human Subjects Act (WMO). This study was approved by the Medical Ethical Committee (METc) of the UMCG (no2021/357). The study has been registered on ClinicalTrials.gov under trial number NCT05440812.

### Statistical analysis

#### Power calculation

Based on previously conducted meta-analyses on the effect of psychosocial interventions [71], behavioural activation treatment [72] and supported internet-based treatment [73] on the social functioning of patients with depression, a medium effect size is expected. The sample size estimation for the RCT was based on a Cohen’s d of 0.5, a *p*-value cut-off (alpha) of 0.05 (Z_alpha_ 1.96) and a power (1-beta) of 80% (Z_beta_ 0.84). The following calculation was used to obtain the number of needed participants per group (n_gr_): N_gr_ = (SD_gr1_ + SD_gr2_/ratio) * ((Z_alpha_ + Z_beta_)/d)^2^, where the ratio between the two groups’ sample sizes (n_gr1_/n_gr2_) was set to 1 (equal group sizes). This calculation resulted in (1 + 1/1) * ((1.96 + 0.84)/0.5)^2^ = 2 * 31.4 = 62.8) an estimated 63 participants needed per group (total sample size = 126). With an expected dropout rate of 10%, we arrived at a total required sample size of 140 ((126/9)*10), with 70 participants per treatment group.

### Data analysis

#### Quantitative analyses

Before the start of the analyses, any missing values will be imputed 20 times using a fully conditional specification [74]. After multiple imputation, all analyses will be conducted in each of the 20 datasets and pooled across imputed datasets [75]. No subjects will be excluded in any phase of the data preprocessing and analyses. All analyses will be conducted according to the intention-to-treat (ITT) principle, and findings will be reported following the Consolidation of the Standards of Reporting Trials (CONSORT) guidelines [76].

The primary outcome in this study is the change in the level of subjective experienced recovery during the intervention period (T0 to T1) and at the six-month follow-up (T0 to T2). The efficacy of the STAIRS-training will be examined by comparing the change in recovery scores between the intervention group and the control group using linear regression models with the treatment group (0 = control; 1 = intervention) as the main independent variable. In subsequent analyses, subgroup differences in the change in the primary outcome will be explored by testing the interaction effects of treatment condition with a range of baseline covariates: age, gender, marital status, employment status, level of education, family composition, number of previous depressive episodes, duration of current depression, first age of onset, and sleeping problems. Intervention effects on the continuous secondary outcomes are analysed using the same approach as for the primary outcome. To investigate the effect of treatment on the relapse rate (during follow-up; T1 to T2), logistic regression will be used with relapse at T2 (1 = yes; 0 = no) as the outcome and the treatment group as the independent variable.

For all estimated linear regression models, assumptions of conditional normality, linearity and homoscedasticity will be checked by inspection of Q‒Q plots and residual plots. In case of assumption violations, outcome variables will be transformed (e.g., natural logarithm) and models rerun. If necessary, different transformations will be applied until the model assumptions are met. Imputation and statistical analyses will be conducted with SPSS and/or R. A two-sided alpha level of 0.05 will be used.

#### Qualitative analyses

Qualitative analyses will be performed according to the Qualitative Analysis Guide of Leuven (QUAGOL) [77]. The analysis process described in the QUAGOL consists of ten stages, in which the data are thoroughly read and reread to reach meaningful concepts, come to a conceptual framework and use the findings to answer the research question. All semi-structured interviews will be audio recorded and transcribed verbatim immediately by the interviewing researcher (DW), and a short report about each patient’s characteristics and the context of the interview will be written in the first stage. This transcript will be read different times by DW and another member of the research team to become familiar with the data. The researchers’ first interpretations of passages and key phrases will be written down. In the second stage, a brief summary will be written of each interview containing the key storyline as a narrative report. For the first five interviews, this will be done independently by two researchers, after which the narrative report will be discussed with another member of the research team. A member check will be done by sending the report to the patient who has been interviewed and asking if they recognize themselves in what has been described [78]. Stage three consists of the development of a conceptual interview scheme in which the patients’ experiences will be replaced by more abstract concepts. This means that important data will be filtered from the interview and clustered into concepts. Next, in stage four, the conceptual interview scheme of each interview will be compared with the interview on which it is based to check if it reflects the most important concepts. If needed, the interview scheme will be adapted, completed or refined. In stage five, the concepts will be compared across interview schemes to identify a list of common concepts. New concepts can be added to this list if needed, and comparable concepts can be merged into one, where appropriate. In stage six, the coding process starts. Here, the concept list will be evaluated and discussed by the research team. The resulting list of concepts will then be transferred as codes to Atlas.ti. In stage seven, all interviews will be read thoroughly again, and significant passages are given a code. Questions regarding the concepts and/or possible necessary changes to the list are noted and discussed in the research team, after which the list may be adapted. After coding, one-third of the interviews will be analysed independently by two researchers in stage eight to check if all citations indeed match the code and to specify the different concepts by adding the context (when, why, under which circumstances, etc., do the concepts appear). In stage nine, all concepts are integrated into a conceptual framework, which will be used in stage ten to systematically describe the findings with regard to the experienced effects, the mechanisms contributing to it and the usability and acceptability of the STAIRS program. These findings will be discussed by the research team to answer the research question.

### Economic evaluation

As part of the RCT, the cost-effectiveness of STAIRS will be evaluated. Analyses will be performed using the societal perspective, as we are interested in all relevant costs, including direct costs (healthcare costs) and indirect costs (productivity loss). Healthcare costs will be calculated by multiplying the healthcare resource use (general practitioner visits, hospital days, etc.) during the last three months, as assessed with the TIC-P, by their reference cost prices as described in the guideline for economic evaluation [79]. The costs for delivering the intervention will be calculated by multiplying the number of hours spent by both trainers per group by the average hourly wage of a clinician and an expert by experience according to the collective employment agreement for Dutch mental healthcare and dividing this by the average number of participants per group. Productivity loss will be calculated by summing the costs of absenteeism and presenteeism. Absenteeism will be calculated using the friction cost method by multiplying the lost working hours in the last month by an average hourly wage. The percentage of work loss due to presenteeism will be calculated by multiplying the percentage of lost productivity while at work (indicated by patients on a 0–10 range) by the number of hours affected. Although productivity loss is expected to be less important in the studied population, as unemployment is relatively high, data on productivity loss will be collected anyway because the intervention under study is also targeted at regaining satisfactory functioning in a (paid or unpaid) job again. All reference cost prices and average hourly wages will be corrected based on inflation rates to represent realistic costs for the present time.

The cost-effectiveness of CAU + STAIRS versus CAU + basic psychoeducation will be analysed by calculating the incremental cost-effectiveness ratio (ICER). For this, the difference in total costs of both interventions (healthcare and productivity costs between T0 and T1/T2, plus the costs of deploying the intervention) will be divided by the difference in effect of both interventions. The incremental costs will be reported per QALY gained as well as per point gained on the I.ROC to provide insights into both the cost-utility and the cost-effectiveness of the STAIRS program.

We expect a significant difference between participants in both interventions in the level of recovering quality of life and consequently in QALYs, favouring the STAIRS-program. Furthermore, a reduction in healthcare and productivity costs is expected for the STAIRS group. As the costs for delivering the STAIRS-training are low, the ICER is expected to be well below the threshold set by the National Health Care Institute, showing that STAIRS is a cost-effective program. If there is no significant difference in the level of recovering quality of life between STAIRS and the control group at the 6-month follow-up (T1), but a positive trend is visible, we will model a future expectation with a best case, conservative case and worst case scenario. Based on these scenarios, the possible long-term cost-effectiveness will be calculated.

## Discussion

To the best of our knowledge, this is the first study to test the efficacy of a program that combines the use of peer support, family support, experts by experience and professional blended care to facilitate the recovery process of MDD patients. The importance of recovery-oriented treatment for patients suffering from severe mental disorders is increasingly recognized [5, 31]. However, in mental healthcare, programs that are designed to promote functional and personal recovery for MDD patients are lacking [80]. To address this gap, the STAIRS-training was developed by a project team consisting of clinicians and experts by experience to promote functional and personal recovery. With this mixed-methods RCT, we aim to evaluate the efficacy of adding this newly developed training to CAU on patients’ level of functional and personal recovery and associated aspects, including symptom severity, relapse, depression-related disabilities, empowerment, self-management skills and experienced control over life. Furthermore, this study aims to investigate possible explanatory mechanisms for the observed effects of STAIRS and to investigate possible differences in outcomes between demographic and clinical subgroups. These respective parts of the study are aimed at gaining insights into *how* the STAIRS-training might contribute to recovery and whether there are person-specific factors that can be used to determine for whom STAIRS is most suitable. Finally, this study aims to evaluate the cost-effectiveness of STAIRS. In this RCT, the intervention group will receive the STAIRS-training on top of CAU. The comparison group will receive CAU, augmented with a limited form of psychoeducation to foster patient engagement with the study. If this study provides evidence in support of the efficacy of the STAIRS-training, further steps can be taken toward implementation of the program as an addition to regular treatment to foster functional and personal recovery in daily practice.

The proposed study has several strengths. The first strength is the use of both quantitative and qualitative methods, where qualitative data serve to enhance the interpretability of the quantitative data [47]. On the one hand, the quantitative data will provide insight into the efficacy of the STAIRS program. On the other hand, the results from the interviews may provide a better understanding of how the program is experienced, which in turn may provide more insight into the mechanisms underlying any effects observed in the quantitative analyses. In addition, the qualitative part allows for the investigation of the usability and acceptability of the program. The second strength is that although the intervention focuses on functional and personal recovery, measurements also encompass clinical recovery. This allows for investigation of the dependencies between the different domains of the recovery process. For instance, if we find that improvement in the level of functional and personal recovery is accompanied by a decrease in (residual) symptom severity and a lower probability of relapse than CAU, this would emphasize the importance of recovery-oriented programs as an addition to more traditional symptom-focused depression treatment. Finally, to the best of our knowledge, the STAIRS-training is the first program for patients with a depressive disorder that combines different elements, which are known to foster functional and personal recovery, based on previous research [31, 32, 46]. By combining peer support, guidance by a clinician and an expert by experience, face-to-face and online facilities (blended care), and involving significant others, we expect the STAIRS-training to facilitate important supportive mechanisms.

This study also has some expected difficulties. First, recruitment may be difficult because of the timing of the interventions in this trial. Patients are recruited during the final phase of their treatment and may therefore be reluctant to participate, as this may feel as a delay of the completion of their treatment. To minimize this risk, we emphasize to patients that STAIRS is an add-on to regular treatment with a specific focus on areas that are considered important by patients themselves in the final phase of treatment. Second, treating clinicians may be reluctant to inform eligible patients about the study because they might have concerns about not being able to end regular treatment during the runtime of the trial. We will therefore emphasize to clinicians that nothing needs to be changed in the current treatment. If the treatment is scheduled for completion during the RCT, this will not change because of participation in this study. Third, because the STAIRS-training is a group intervention, patients assigned to the STAIRS-training have to wait before they can start until enough subsequent patients are randomized to the STAIRS condition to fill up a group. If recruitment is slow, a trade-off may be necessary between reducing the waiting time for patients on the one hand and achieving the target group size on the other. To prevent patients from having to wait too long, a group can start when at least three patients are assigned. However, group dynamics in smaller groups may differ from those in larger groups, which may lead to different results. We will therefore compare the results of different groups based on group size to investigate possible differences. As the group sizes of existing group training programs within mental health care also differ, insight into possible differences can also be helpful when implementing the STAIRS program in daily practice. Last, some patients may be reluctant to join face-to-face group meetings because they feel too exposed or have practical objections, such as excessive travel time. To give these patients the opportunity to participate, the STAIRS program can also be offered online. However, efficacy, patient experiences and cost-effectiveness may differ between the face-to-face and online version of the program. When analysing the data, we will use methods that can account for possible differences between groups, while taking into account a control condition that is ungrouped [81]. Furthermore, the results from the semi-structured interviews will be compared between patients who followed the STAIRS program face-to-face and those who followed the program online. Finally, in addition to overall cost-effectiveness, the cost-effectiveness per program version will also be assessed and compared.

If the results from this study demonstrate the efficacy and cost-effectiveness of the STAIRS-training, broader implementation of the STAIRS program as an addition to the existing provision of mental healthcare for MDD patients may be a useful and promising next step. To determine the optimal way to implement the program, patients’ evaluations of the acceptability and usability of the training are likely to provide very helpful insights. In addition, these evaluations might also be helpful for other researchers who seek to develop other recovery-oriented interventions. The results from this study will be communicated via several publications.

### Supplementary Information

**Additional file 1: Appendix 1** **and** **Appendix 2.** 

## Data Availability

The datasets generated and/or analysed during the current study are not publicly available due to personal data in the datasets. Only the project team members will have access to the dataset. The processed, pseudonymized data are available from the principal investigator on reasonable request if the research question falls within the scope of the informed consent.

## Abbreviations

MDD: Major Depressive Disorder
STAIRS: Storytelling and Training to Advance Individual Recovery Skills
CAU: Care As Usual
RCT: Randomized Controlled Trial
QALY: Quality Adjusted Life Year
UMCG: University Medical Center Groningen
UCP: University Center Psychiatry
IDS-SR: Inventory of Depressive Symptomatology-Self Report
I.ROC: Individual Recovery Outcome Counter
RAS-DS: Recovery Assessment Scale-Domains and Stages
SDS: Sheehan Disability Scale
MDE: Major Depressive Episode
MINI-S: Mini International Neuropsychiatric Interview-Simplified
NEL: Netherlands Empowerment List
SMAS: Self-Management Ability Scale
TiC-P: Treatment inventory of Cost in Patients with psychiatric disorders
SF-HLQ: Short Form Health and Labour Questionnaire
ReQol: Recovering Quality of Life
SRM: Standardized Response Mean
ReQol-UI: Recovering Quality of Life Utility Index
COREQ: COnsolidated criteria for Reporting Qualitative studies
DMP: Data Management Plan
GDPR: General Data Protection Regulation
WMO: Wet Medisch-wetenschappelijk onderzoek, Dutch Medical Research Involving Human Subjects Act
METc: Medisch Ethische Toetsings commisie, Medical Ethical Committee
ITT: Intention To Treat
CONSORT: CONsolidation of the Standards of Reporting Trials
QUAGOL: Qualitative Analysis Guide of Leuven
ICER: Incremental Cost-Effectiveness Ratio
