# Supplementary material for: A blended module (STAIRS) to promote functional and personal recovery in patients with a major depressive disorder in remission: study protocol of a concurrent mixed methods randomized controlled trial
Source: BMC Psychiatry. 2023 Oct 7;23:727. doi: 10.1186/s12888-023-05213-w (PMC10560411; doi:10.1186/s12888-023-05213-w)
Supplement: Supplementary file 1 — Additional file 1: Appendix 1 and Appendix 2. [file 12888_2023_5213_MOESM1_ESM.pdf]

Handvatten voor herstel: Structuur

De depressie is (deels) achter de rug, en nu? De meeste mensen willen hierna zo snel mogelijk het leven weer oppakken. Vanuit de ervaringen van anderen is bekend dat het soms best lastig kan zijn om weer controle te ervaren over je eigen leven en dat het tijd kost om dit te bereiken.

De fase na de depressie waarin een nieuw evenwicht gezocht wordt noemen we de *persoonlijk herstel* fase. Er zijn grofweg drie verschillende gebieden waarvoor in deze fase aandacht nodig is:

1. Het opnieuw vormgeven van hoe je dagelijks leven eruit ziet.
2. Het opnieuw vormgeven van de verschillende contacten die je hebt en de rol die je daarin aanneemt.
3. Het opnieuw uitvinden van hoe je jezelf ziet.

In het schema hieronder zie je de verschillende gebieden waarvoor aandacht nodig is om goed te kunnen herstellen. In de depressiebehandeling is veel aandacht voor het verminderen van de depressiesymptomen, wat we symptomatisch herstel noemen. Onderdeel van de behandeling is vaak ook aandacht voor dagelijks- en maatschappelijk functioneren, maar dan met name gericht op hoe dit van invloed is op de depressiesymptomen. Naast het belang voor symptomatisch herstel, zijn deze gebieden echter ook belangrijk voor het vinden van een nieuw evenwicht in je leven (waarmee weer grip ervaren wordt). Daarnaast spelen vragen rondom veranderingen in hoe je jezelf bent gaan zien sinds de depressie hierin een belangrijke rol. Om hierbij te helpen krijg je gedurende de komende 7 weken drie keer de informatiebrief 'Handvatten voor herstel', waarin telkens bij een van deze gebieden wordt stil gestaan.

Deze eerste Handvatten voor herstel staat stil bij het thema structuur. Dit is een belangrijk thema als het gaat om het herstellen van het dagelijks functioneren.

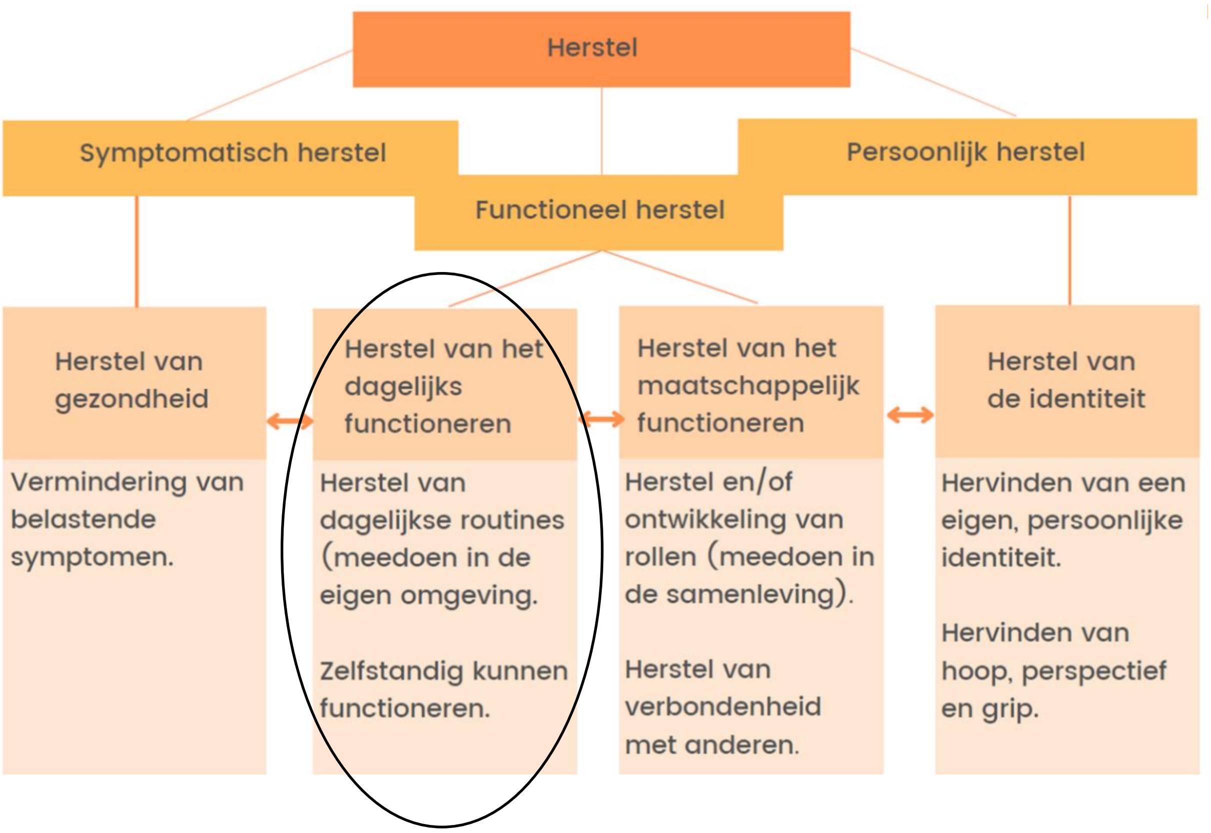

Structuur

Een depressie heeft veel impact op je leven en kan de manier waarop je je dagen invult flink overhoop gooien. Nadat de symptomen van een depressie niet meer in sterke mate aanwezig zijn is er ruimte om naar de toekomst te kijken. Voor veel mensen betekent dit in eerste instantie uitzoeken hoe het dagelijks leven weer ingevuld gaat worden. Het opnieuw vinden van structuur in je leven is belangrijk, maar kan ook behoorlijk ingewikkeld zijn. Allerlei vragen kunnen hierbij een rol spelen. Welke blijvende gevolgen heeft de depressie op mijn leven? Wat wil ik graag (weer) gaan doen? Wat is hierin haalbaar? Waar moet ik rekening mee houden? Hieronder staan een aantal concrete handvatten die je kunnen helpen bij het herstellen van structuur in je dagelijkse leven.

Handvatten:

Schrijf alle gevolgen van jouw depressie die je tegenkomt in je dagelijks leven op. Het kan hierbij helpen om een week lang aan het einde van elke dag op te schrijven welke invloed de depressie gehad heeft op wat je wel/niet hebt gedaan. N.B. denk eraan dat gevolgen niet alleen negatief hoeven te zijn. Er kunnen ook dingen veranderd zijn in je dagelijks leven waar je prima tevreden mee bent.
Alles tegelijk aanpakken blijkt vaak lastig te zijn. Kies daarom eerst 1 van de gevolgen uit waar je mee aan de slag wil. Welke hulpmiddelen kun je inzetten om je doel te bereiken?

Maak een overzicht van hoe je week eruit ziet. Schrijf hiervoor per dag op wat je doet in de ochtend, middag en avond. Sta hierna stil bij de volgende vragen:

- Welke factoren spelen een rol bij het indelen van je week?
- Voel je je vrij om je week zo in te plannen als je wil? Zo niet, wat speelt hierin mee?
- Zitten er mogelijk teveel of te weinig activiteiten in je week?

Maak hierna nog een overzicht, maar dan van je ideale week. Als je volledig zelf kon kiezen, hoe zou je week er dan uitzien? Vergelijk nu beide schema’s en ga op zoek naar mogelijke verschillen en vraag jezelf af wat je kunt doen om dichter bij je ideale weekschema te komen. N.B. het kan helpen om anderen hierin mee te laten denken. Denk er ook aan (een paar) kleine oplossingen soms makkelijker te realiseren zijn dan een grote oplossing. Elke stap dichter naar je gewenste weekschema is winst!

Houd er rekening mee dat veranderingen met vallen en opstaan gaan. Soms lukt iets heel goed en soms ineens niet meer. Door te blijven proberen wordt het uiteindelijk wel steeds beter. Deel wat je doet (of van plan bent te gaan doen) met anderen, zodat ze je erbij kunnen helpen.

Denk ook aan voldoende ontspanningsmomenten in je weekschema. Waar krijg jij energie van en wat kost jou energie? N.B. niet van alles wat leuk is laad je ook op. Als je weet waar jij van ontspant, geef dit dan een goede vaste plek in je weekschema.

Wil je zien welke rol het thema structuur bij anderen speelt, hoe ze hiermee omgaan en/of op welke manier je hierover in gesprek kunt gaan met anderen? Neem dan eens een kijkje op https://allesgoed.org/thema-filter/ en klik de tegel ‘Structuur’ aan om verhalen te zien die gaan over (het belang van) structuur.

Handvatten voor herstel: Verbinding maken

Wat is er bij jou veranderd in het contact met anderen? Veel mensen ervaren na een depressie dat het lastig kan zijn om weer helemaal mee te kunnen doen. Bij het vinden van een nieuwe balans in je leven is het daarom goed om aandacht te hebben voor de veranderingen die er zijn in het contact met anderen.

Het opnieuw vormgeven van de verschillende contacten die je hebt en de rol die je daarin aanneemt is een van de gebieden die bij persoonlijk herstel horen. In het schema hieronder zie je nogmaals wat de verschillende gebieden zijn waar aandacht voor nodig is om te herstellen.

Met de vorige Handvatten voor herstel heb je gewerkt aan het herstel van je dagelijks functioneren door bezig te gaan met structuur in je leven. Deze tweede Handvatten voor herstel staat stil bij het thema verbinding maken. Dit is een belangrijk thema als het gaat om het herstellen van het maatschappelijk functioneren.

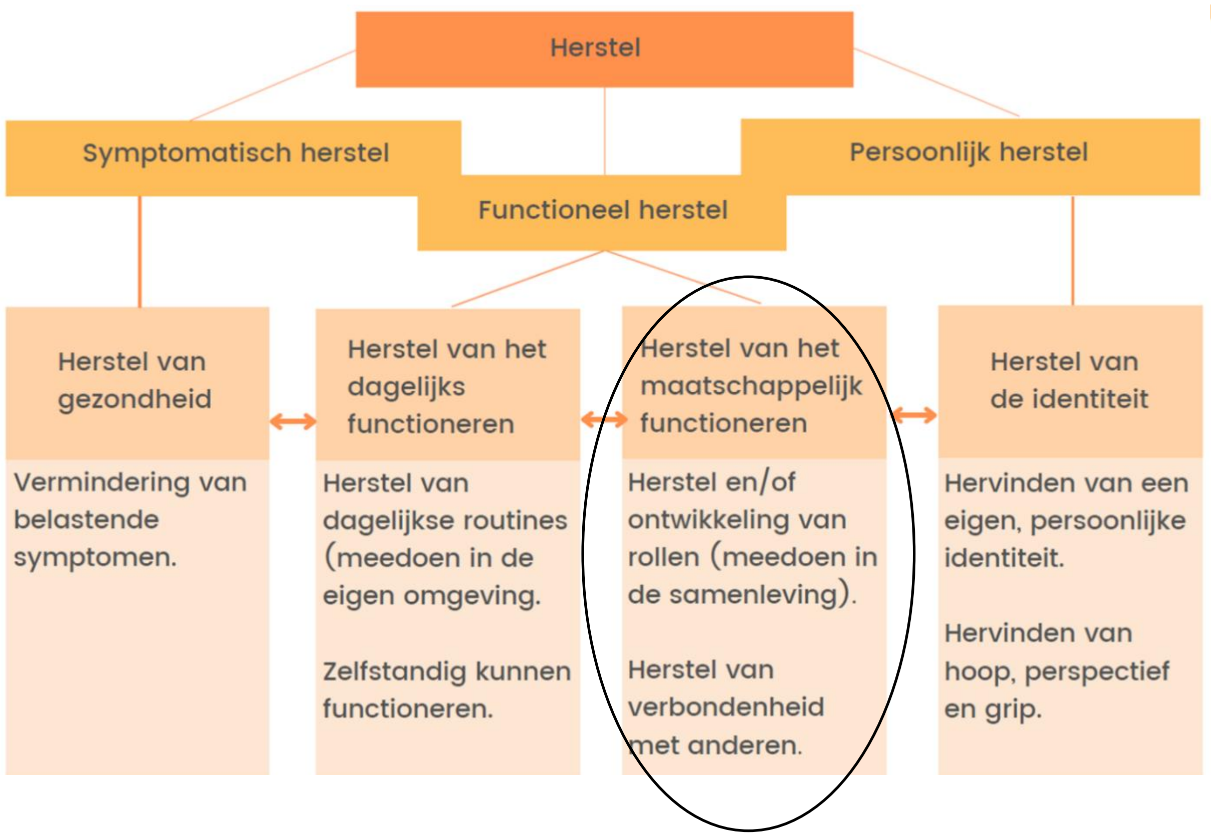

Verbinding maken met anderen

Contact met anderen en mee kunnen doen zijn belangrijke aspecten in ieders leven. Een depressie kan hier veel invloed op hebben. Je kunt hierbij denken aan veranderingen in het contact dat je hebt met je partner, je gezin, met collega's of met vrienden. Veel mensen hebben het gevoel dat er anders met hen omgegaan wordt sinds de depressie. Misschien zetten anderen nu wel vraagtekens bij wat je aankan, terwijl dit eerder nooit zo was. Of merk je dat het lastig is het gesprek aan te gaan met anderen. Het beeld dat jijzelf en anderen van jou hebben kan door de depressie veranderd zijn. Ook als de depressie zelf een stuk minder aanwezig is, kan dit beeld nog blijven bestaan. Onderzoek heeft laten zien dat negatieve beeldvorming (stigma) rondom het hebben van een depressie veel invloed kan hebben op hoe er met je omgegaan wordt, welke mogelijkheden je wel/niet krijgt en hoe je jezelf ziet. Ook als het goed bedoeld is, bijvoorbeeld doordat anderen je in bescherming willen nemen, kan het onderlinge contact negatief veranderen. Contact met anderen en mee kunnen doen met je omgeving zijn belangrijke aspecten in je herstel. Het is daarom goed hierbij stil te staan en er actief mee aan de slag te gaan en te blijven.

Handvatten:

Wat is er bij jou veranderd in het contact met anderen?<br>Sta een week lang stil bij de contacten die je hebt gehad en hoe deze zijn verlopen. Hiervoor kun je aan het einde van elke dag opschrijven met wie je die dag contact hebt gehad en hoe dit is verlopen.<br>Er zijn verschillende vragen die hierbij kunnen helpen: Hoe eerlijk ben ik geweest over hoe het met me gaat en wat daar invloed op heeft? Waar ben ik blij mee in het contact dat ik vandaag had? Wat mis ik in het contact met mijn omgeving? Welk contact vind ik lastig? Tegen welke moeilijkheden loop ik aan in het contact met anderen en wat doe ik op zo'n moment? Welke dingen doe ik zelf in lastige situaties die misschien niet zo handig of helpend zijn?
Zet de namen van mensen met wie je het gevoel hebt dat het contact veranderd is door de depressie op een rij. Kies hier iemand uit waarmee je graag meer/anders verbinding wil hebben. Schrijf hiervoor eerst in steekwoorden op hoe het contact was voor de depressie, tijdens de depressie en na de depressie.<br>Kijk goed naar het overzicht dat je gemaakt hebt en probeer helder te krijgen wat precies voor jou de kern van de verandering is. Vraag jezelf af wat het aandeel van de ander is geweest en wat jouw aandeel was in deze verandering. Hierna kun je met de ander in gesprek gaan over deze verandering bij jullie beiden en wat je graag wil in het contact. Let er hierbij op dat je de ander niet beschuldigd, maar dat je vanuit jezelf aangeeft hoe je het ervaart en wat je graag wil.
Wil je een praktisch hulpmiddel bij het bespreekbaar maken van (de gevolgen van) je depressie met iemand die belangrijk voor je is? Het allesgoed kaartspel is met dit doel ontwikkeld en kan besteld worden via: https://allesgoed.org/alles-goed-kaartspel/
Veel mensen vinden het helpend om ervaringen van lotgenoten te horen.<br>Ervaringen van anderen met de invloed van een depressie op hun relatie zijn te zien op https://allesgoed.org/hoe-beinvloedt-depressie-je-relaties/<br>Wil je voorbeelden zien van anderen die in gesprek gaan over hun depressie? Kijk dan eens op https://allesgoed.org/hoe-praat-je-over-een-depressie/

Handvatten voor herstel: In balans blijven

Hoe zie jij jezelf sinds de depressie? Een depressie kan de manier waarop je jezelf ziet flink veranderen. Dingen waarvan je eerder dacht dat je die prima kon of waar je veel plezier in had roepen nu misschien twijfels op. Om weer grip te kunnen ervaren op je leven is het goed om hierbij stil te staan en voor jezelf een nieuw realistisch beeld te krijgen van wie je bent en wat je kan.

Het vinden van een nieuw beeld van jezelf dat recht doet aan wie je bent is een van de gebieden die bij persoonlijk herstel horen. In het schema hieronder zie je nogmaals wat de verschillende gebieden zijn waar aandacht voor nodig is om te herstellen.

Met de vorige Handvatten voor herstel heb je gewerkt aan herstel van het maatschappelijk functioneren door bezig te gaan met het contact dat je hebt met anderen. Deze derde Handvatten voor herstel staat stil bij het thema <u>in balans blijven</u>. Om een nieuwe balans in je leven te vinden en deze vast te houden is het heel belangrijk om een goed beeld te hebben van jezelf. Een depressie kan dit beeld sterk negatief beïnvloeden, waardoor je een stuk minder grip en hoop ervaart. Het is daarom goed om aandacht te hebben voor het vinden van een nieuw en realistisch beeld van jezelf.

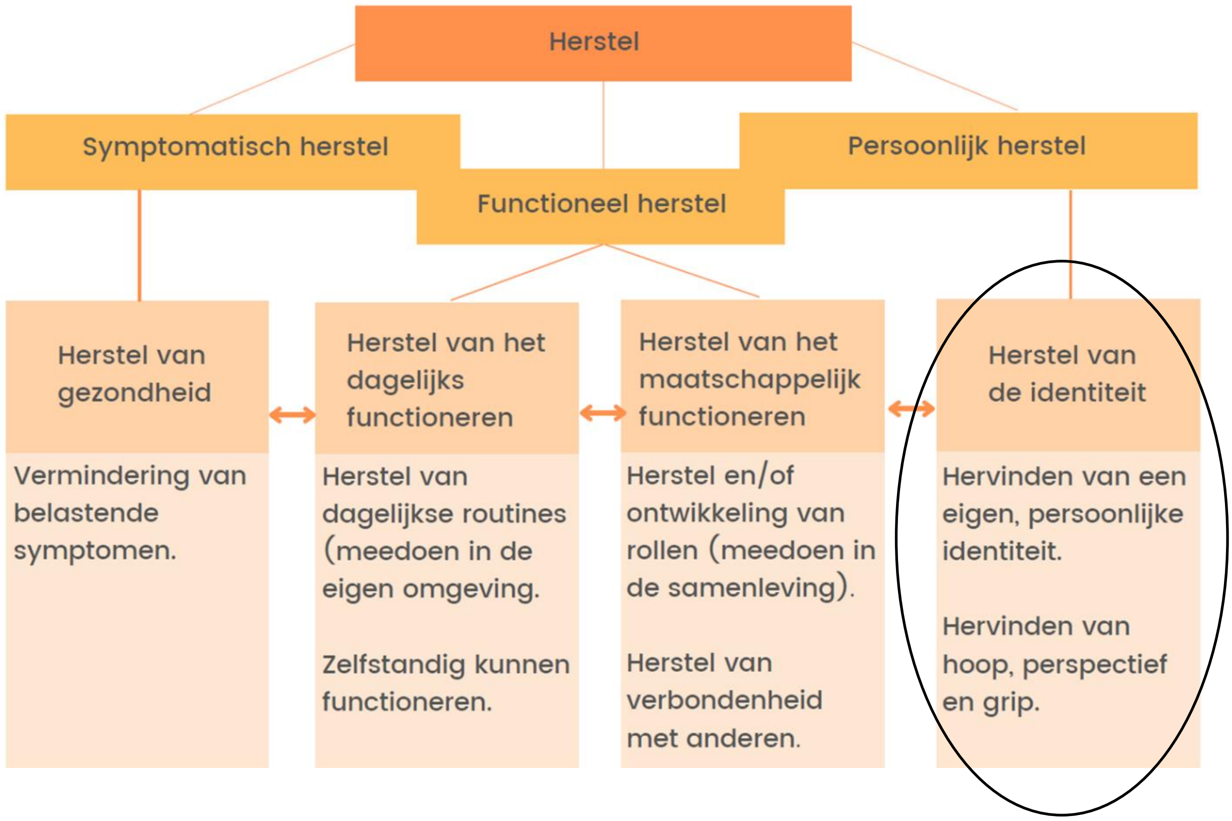

In balans blijven

Uiteindelijk gaat het er bij herstel om dat je een nieuw evenwicht vindt in je leven waar je tevreden mee bent. Dit betekent niet dat je leven weer zo moet zijn als voor de depressie, maar wel dat je voldoende grip ervaart om met de gevolgen om te kunnen gaan. Hiervoor is een realistisch zelfbeeld van belang en zicht op wat je wel en niet belangrijk vindt in het leven. Hoe kijk jij tegen jezelf aan sinds de depressie? Waar ben je goed in en hoe kun je ervoor zorgen dat je controle houdt in situaties die lastig voor je zijn?

Handvatten:

Hoe zie jij jezelf sinds de depressie?<br>Schrijf op een vel papier aan de linkerkant in steekwoorden hoe je jezelf zag voor de depressie en aan de rechterkant hoe je jezelf ziet na de depressie. Wat is voor jou de belangrijkste verandering? Voor veel mensen blijkt dat het beeld dat ze van zichzelf hebben niet realistisch is. Bovendien is het afhankelijk van de situatie of en hoe een eigenschap naar voren komt en kunnen eigenschappen veranderen. Om hier meer zicht op te krijgen kan het helpen om bij de kenmerken/eigenschappen die je bij jezelf vindt passen concrete situaties op te schrijven die laten zien dat je ook anders kan.<br>NB. Vraag ook eens aan iemand die belangrijk voor je is hoe hij/zij jou ziet en in hoeverre dit veranderd is door de depressie.
Waar heeft de depressie voor jou gevoel de meeste impact gehad? Om hier meer zicht op te krijgen kun je een sociogram maken. Schrijf hiervoor je eigen naam in het midden van een papier en hieromheen de verschillende contacten die je hebt. Onder elk contact zet je vervolgens de rol die je hebt t.o.v. dit contact (bijv. partner, ouder, vriend, etc.). Kies vervolgens een rol die voor jou belangrijk is en waar je iets in wil veranderen. Vragen die kunnen helpen om hieraan te werken zijn bijvoorbeeld: hoe wil je de rol graag invullen? Hoe wil je dat de ander jou ziet? Waarom is deze rol zo belangrijk voor je? Wat heb jij in huis dat je zou willen laten zien en/of waar je gebruik van kan maken? Ook hier geldt dat het helpend kan zijn om het gesprek hierover aan te gaan met degene waar de rol mee te maken heeft.
Wat is voor jou belangrijk in je leven? Het is makkelijker een gezonde balans in je leven te vinden als je dingen doet die aansluiten bij wat jij belangrijk vindt. Kijk eens naar een lijst met waarden (bijv. via https://www.movisie.nl/sites/movisie.nl/files/2018-04/Waardenlijst-bij-waardenoefening.pdf) en noteer de waarden die voor jou belangrijk zijn. Bedenk vervolgens welke dingen jij al doet die aansluiten bij deze waarde(n) en welke dingen er niet aan bijdragen. Wat zou je nog meer kunnen doen dat goed past bij de waarden die jij belangrijk vindt?
Om in balans te blijven is het belangrijk alert te zijn op signalen dat het minder goed met je gaat. Mocht je deze nog niet op een rijtje hebben, maak hier dan een overzicht van (samen met iemand die hier mede alert op kan zijn). Bedenk ook wat je kunt doen als het minder goed gaat. Door hier van te voren alvast over na te denken is het makkelijk te doen mocht het nodig zijn.
Wil je zien welke rol het thema in balans blijven bij anderen speelt, hoe ze hiermee omgaan en/of op welke manier je hierover in gesprek kunt gaan met anderen? Neem dan eens een kijkje op https://allesgoed.org/thema-filter/ en klik de tegel 'Grip' aan om verhalen te zien die gaan over het krijgen en behouden van grip in je leven na een depressie.
